# Identification of Novel Genomic Regions and Superior Alleles Associated with Zn Accumulation in Wheat Using a Genome-Wide Association Analysis Method

**DOI:** 10.3390/ijms21061928

**Published:** 2020-03-11

**Authors:** Zhengfu Zhou, Xia Shi, Ganqing Zhao, Maomao Qin, Maria Itria Ibba, Yahuan Wang, Wenxu Li, Pan Yang, Zhengqing Wu, Zhensheng Lei, Jiansheng Wang

**Affiliations:** 1 Wheat Research Institute, Henan Academy of Agricultural Sciences, Zhengzhou 450002, China; zhouzf215@163.com (Z.Z.); mrshi0614@126.com (X.S.); qmm1988630@126.com (M.Q.); wyh.best@163.com (Y.W.); tinbingye@163.com (W.L.); ypang0916@163.com (P.Y.); wzhqfy@163.com (Z.W.); 2 College of Chemistry and Environment Engineering, Pingdingshan University, Pingdingshan 467000, China; zgq1118@163.com; 3 Global Wheat Program, International Maize and Wheat Improvement Center (CIMMYT), Mexico, D.F. 06600, Mexico; M.IBBA@cgiar.org; 4 College of Life Sciences, Zhengzhou University, Zhengzhou 450001, China

**Keywords:** wheat, zinc accumulation, GWAS, genetic loci, superior and inferior alleles

## Abstract

Micronutrient deficiencies, and especially zinc (Zn) deficiency, pose serious health problems to people who mainly depend on cereal-based diets. Here, we performed a genome-wide association study (GWAS) to detect the genetic basis of the Zn accumulation in wheat (*Triticum aestivum* L.) grains with a diversity panel of 207 bread wheat varieties. To uncover authentic quantitative trait loci (QTL) controlling Zn accumulation, the varieties were planted in three locations. In total, 29 unique loci associated with Zn grain accumulation were identified. Notably, seven non-redundant loci located on chromosomes 1B, 3B, 3D, 4A, 5A, 5B, and 7A, were detected at least in two environments. Of these quantitative trait loci (QTL), six coincided with known QTL or genes, whereas the highest effect QTL on chromosome 3D identified in this study was not reported previously. Searches of public databases revealed that the seven identified QTL coincided with seven putative candidate genes linked to Zn accumulation. Among these seven genes, *NAC domain-containing protein* gene (*TraesCS3D02G078500*) linked with the most significant single nucleotide polymorphism (SNP) AX-94729264 on chromosome 3D was relevant to metal accumulation in wheat grains. Results of this study provide new insights into the genetic architecture of Zn accumulation in wheat grains.

## 1. Introduction

Over two billion of the world’s population suffer from diseases associated with mineral and essential vitamin deficiencies [1,2,3]. Zinc deficiency in particular affects ~17% of the people worldwide, mainly from developing countries in Asia and Africa. The insufficient absorption of such micronutrient causes severe problems to human health, affecting the vital growth and the cognitive development in young children, and resulting in excessive weight loss, depression, psychosis, diarrhea, impaired growth and development, and altered reproductive capacity for child-bearing female [4,5,6]. In addition, zinc, as one of the eight essential micronutrients for plant, is an important constituent of several enzymes and proteins regulating DNA transcription, protein, nucleic acid, carbohydrate, and lipid metabolism [7,8,9]. For instance, zinc as a cofactor is needed for the activation of some of the enzymes involved in the creation of the plant growth hormone auxin and the formation of chlorophyll [10,11]. Zinc also plays an essential role on the structural stability of certain proteins, such as transcription factors containing Zn-finger domains, and deficiency of this mineral might result in significant reduction in crop yields and quality [12]. Therefore, both plants and humans require an optimum intake of zinc for their normal physiological and biochemical activity.

Hexaploid wheat (*Triticum aestivum* L.) is one of the most important cereal crops worldwide and its products provide ~20% of the energy and proteins in human diet [13,14]. Because of the central role that wheat has in human nutrition, wheat biofortification could serve as a cost-effective and efficient strategy to alleviate Zn deficiencies, especially in low income countries where the majority of people rely on a cereal-based diet [15,16,17]. For this reason, improving zinc accumulation in wheat grains is an important target for wheat breeding.

Genetic mapping of quantitative trait loci (QTL) could facilitate the understanding of the genetic basis of a complex quantitative trait such as zinc accumulation in wheat grains, thus providing a theoretical basis to guide crop mineral nutrients improvement [18,19]. Using different bi-parental populations derived from both diploid, tetraploid, and hexaploid wheat varieties, several QTL associated with variation in Zn grain content were identified on chromosomes 1A, 2A, 2B, 3D, 4B, 6A, 6B, and 7A [20,21,22,23,24,25]. However, the QTL identified using bi-parental populations have a low resolution and are restricted to the genetic variation identified in the two parents used to obtain the mapping population. In contrast, by performing a genome-wide association study (GWAS), it would be possible to greatly improve the QTL resolution by using a more representative and varied gene pool [26]. This analysis in fact allows the detection of non-random associations between genotype and phenotype in a group of individuals in a germplasm panel [27] and, at present, has been widely applied to detect genetic variants related to complex agronomic traits in different plant species [28]. However, to the best of our knowledge, only two GWAS studies have been performed in wheat to identify QTL associated with Zn grain content. Specifically, in the study conducted by Velu et al. [29] were identified major effect QTL on the 2 and 7 group chromosomes using a population of 330 spring common wheat varieties. In the study conducted by Alomari et al. [30], markers associated with greater Zn accumulation were identified across the majority of the chromosomes but were concentrated on chromosomes 3B and 5A.

Nevertheless, despite the number of genetic studies performed on wheat to identify the genetic regions associated with Zn grain content, the molecular mechanisms behind Zn accumulation in wheat grain remain unclear and no molecular markers which could facilitate the breeding for Zn content have been identified. The use of a different set of germplasm and of higher resolution genotyping techniques could help to shed light on the mechanisms behind the accumulation of this mineral in wheat grain. In the present study we conducted a GWAS on a diverse panel of 207 common wheat cultivars grown in three different environments and genotyped using the high-resolution Wheat Breeders 660 K Axiom^®^ array. The main purposes of this study were to: (1) investigate novel significant molecular markers or loci associated with potential candidate genes for Zn grain accumulation; (2) provide useful information to better understand the molecular and genetic mechanisms of Zn accumulation in common wheat, and (3) identify germplasms with higher than average Zn grain content that could be used as donors for biofortification breeding. Results of this study could contribute to the development of strategies to improve the nutritional quality of wheat.

## 2. Results

### 2.1. Variation in Zn Contents among the Wheat Lines

**Figure 1:**
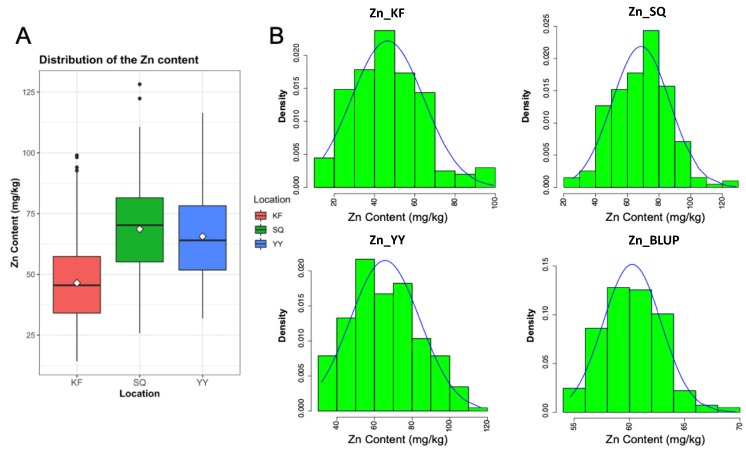
Distribution of the grains zinc concentrations measured in the wheat association mapping population. (**A**) Boxplot of zinc grain concentration in Kaifeng (KF), Shangqiu (SQ), and Yuanyang (YY) location. (**B**) Distribution of the zinc grain concentration in the individual environments (including the combination of three locations, BLUP) among the 207 hexaploid wheat accessions.

Across the three environments, a continuous and wide variation of Zn grain concentrations was observed. As reported in Figure 1A, on average, Zn concentrations in wheat grains were higher in the Shangqiu (SQ) location where reported values ranged from 25.83 to 128.22 mg/kg (average 68.63 mg/kg). The second highest Zn grain values were observed in the Yuanyang (YY) location with average values of 65.63 mg/kg and a range of 31.87–116.47 mg/kg. Drastically lower values were recorded in Kaifeng (KF) (average Zn content of 46.45 mg/kg), where Zn grain concentration values ranged from 14.24 to 99.00 mg/kg. Similar values were obtained after combining the data from the three environments through BLUPs (average 60.24 mg/kg; range 54.65–69.65 mg/kg). In all cases (three environments and BLUPs), the values of Zn grain content followed an approximately normal distribution (Figure 1B, Table 1) suggesting that this diversity panel is suitable for conducting GWAS for wheat grain Zn concentrations.

### 2.2. Screening of SNPs and Statistical Model Selection

**Figure 2:**
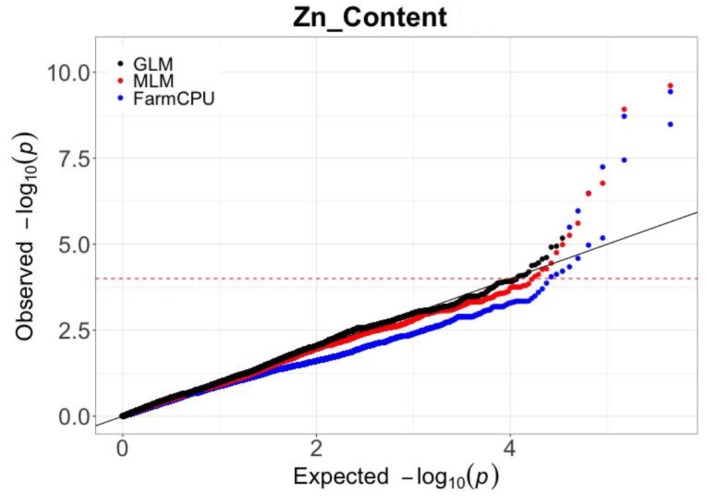
Quantile–quantile (QQ) plots for wheat grains zinc accumulation using three models. Black dots, GLM model; Red dots, MLM model; Blue dots, FarmCPU model; The black line indicates the expected values; The red dotted line indicate the −log_10_(*p*) threshold of 4.0.

All the 207 lines included in the association panel were genotyped using the wheat 660K SNP array. After filtering the markers for minor allele frequency >0.05 and missing data of <10%, were obtained a total of 244,508 SNPs which were used for the following analysis (Figure S1). The GWAS analysis was performed using three different models: (1) GLM (only controls population structure), (2) MLM (accounts for population structure and relative kinship), and (3) FarmCPU (alternately uses fixed and random effect models to improve statistical power). The analysis was performed separately for the three different environments and for the combined data. The quantile–quantile plots (QQ plot) obtained from each analysis were then compared and used to identify the optimal statistical model for the GWAS analysis. As could be seen in Figure 2, the GLM model exhibited a high risk of type I errors (false positives) whereas the FarmCPU model was too strict and resulted in higher type II errors (false negatives). Compared with the GLM model and FarmCPU, the MLM model appeared to be the best choice being able to significantly reduce both the type I and type II errors. For these reasons, only the results obtained with the MLM model will be presented.

### 2.3. Genome-Wide Association Study with Individual SNP Markers

**Figure 3:**
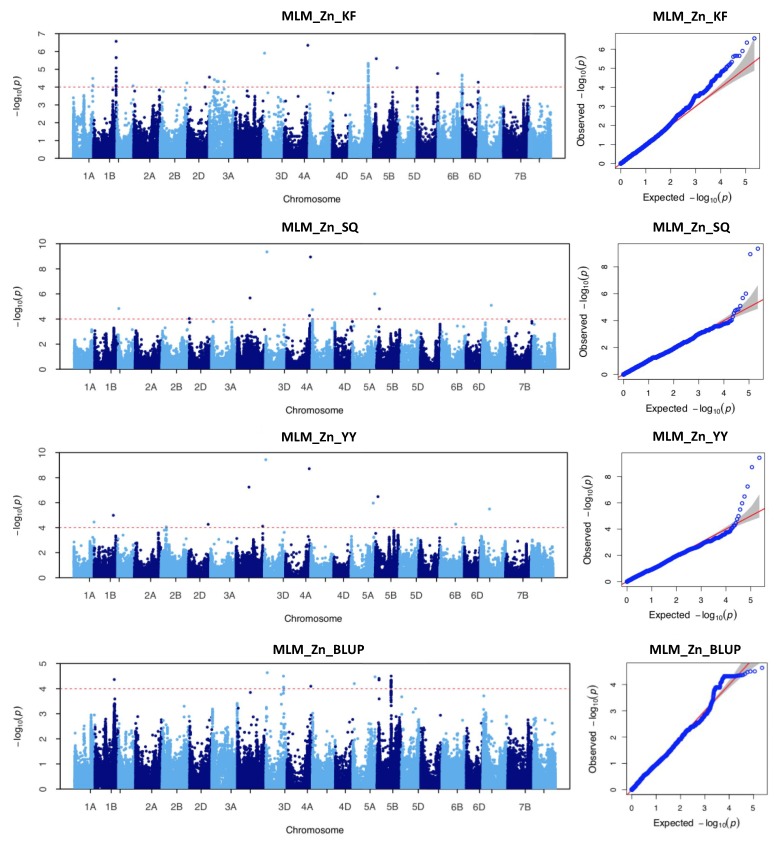
Manhattan and QQ plots of genome-wide association scan for wheat grains zinc accumulation in the 207 wheat association mapping population using the mixed linear model (MLM) in GAPIT software. Each dot represents a SNP. The horizontal dashed line represents the significant threshold −log_10_(*p*) equaling 4.0. The SNPs above the red dotted line are all significantly associated with Zn grain variation. Quantile–quantile plots resulting from genome-wide association study (GWAS) results using MLM model for wheat grains zinc accumulation in different environments. MLM_Zn_YY, mixed linear model results obtained from the Yuanyang location; MLM_Zn_KF, mixed linear model results obtained from the Kaifeng location, MLM_Zn_SQ, mixed linear model results obtained from the Shangqiu location, MLM_Zn_BLUP mixed linear model results obtained from the BLUP location.

**Figure 4:**
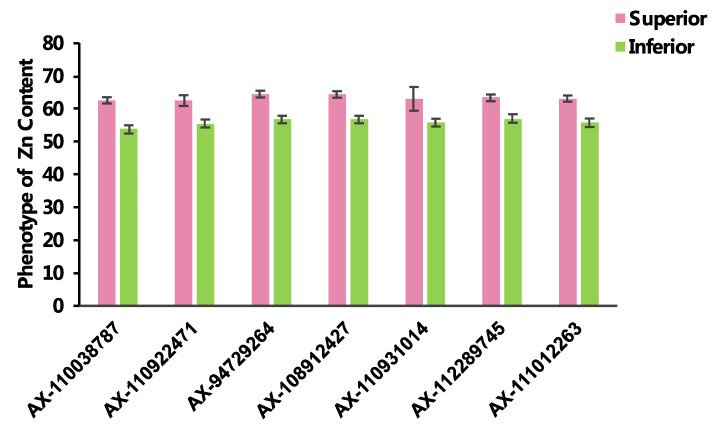
The phenotypic performance in cultivars with superior alleles and inferior alleles in seven repetitive significantly SNPs loci for grains zinc accumulation. Pink bars represent superior alleles phenotype value; Green bars represent superior alleles phenotype value.

From the GWAS analysis, a total of 125 markers were significantly associated (of *p* ≤ 1.0 × 10^−4^) with variation in Zn grain content (Table S2). The identified markers could be categorized into 29 non-redundant QTLs distributed across the whole genome (Table 2). Among the 125 SNPs, 73 were detected using the phenotypic values in individual environments and 52 were identified using the combined data across the three environments (BLUP). Briefly, ten significant SNPs were associated with Zn content variation in the SQ location. They were located on chromosomes 1D, 2D, 3B, 3D, 4A, 4B, 5A, 5B, 7A, and the phenotypic variation explained (PVE) by each SNP ranged from 10.70% to 24.55% with a mean of 15.12%. In the KF location were identified 66 significant SNPs located on chromosomes 1A, 1B, 1D, 2B, 2D, 3A, 3D, 4A, 5A, 5B, 6A, 6B, 6D. Each SNP explained from 9.11% to 15.44% of the phenotypic variation, with a mean of 10.70%. Similarly, in the YY location were identified 12 significant SNPs on chromosomes 1A, 1B, 2B, 2D, 3B, 3D, 4A, 5A, 5B, 6B, 7A, each explaining from 11.37% to 24.77% of the phenotypic variation, and a mean of 15.57%. When using the combined data were identified 52 significant SNPs which, from the three individual locations, were detected only on few chromosomes (1B, 3D, 4A, 5A, and 5B), and explained between 13.04% to 14.40% of the observed variation in Zn grain content (Figure 3, Table 2). Among the 125 significant SNPs, seven were identified in at least two environments (Table 2 and Table 3). Among them, SNPs AX-94729264 and AX-108912427, located respectively on chromosomes 3D and 4A, were significantly associated with variation in Zn content in all the four locations (including BLUP) and exhibited average PVE values ranging from 19.37% and 18.58%, respectively. The SNP AX-112289745 on chromosome 5B (mean PVE of 14.25%) was significantly associated with Zn contents in three locations. Differently, the SNPs AX-110038787, AX-110922471, AX-110931014, and AX-111012263, located respectively on chromosomes 1B, 3B, 5A, and 7A, were identified in two environments and were associated with average PVE values of 13.65%, 16.91%, 15.74%, and 14.01%, respectively (Table 2). All these SNPs that appeared to be consistently associated with variation in Zn grain content in at least two environments could be considered as stable loci for the regulation of Zn accumulation in wheat grains. For each of the seven SNPs that were associated with Zn grain content variation in at least two environments, an ANOVA analysis was then performed. For all the SNPs, the allelic variants associated with higher and lower Zn grain content were identified and the results of the statistical analysis showed that there is a significant difference in Zn grain content between the accessions with the superior alleles and the accessions with the inferior alleles in all or some of the environments (Figure 4, Table 4). Of all cultivars surveyed, accessions with superior alleles displayed higher Zn accumulation in wheat grains than accessions with inferior alleles.

### 2.4. Haplotypes Associated with Zn Grain Content

**Figure 5:**
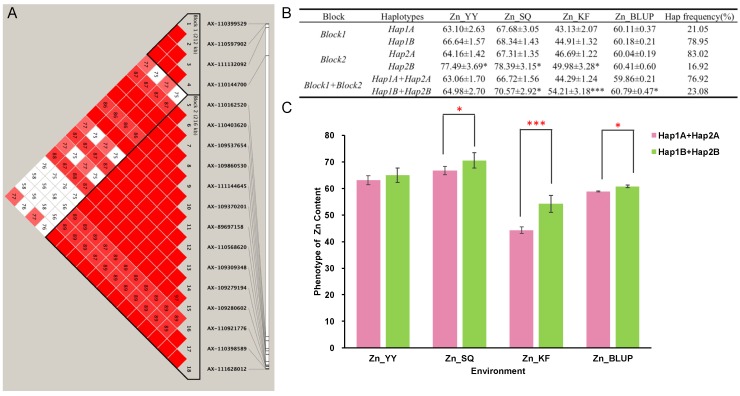
Haplotype analysis for the significant SNPs on chromosome 1B for wheat grains zinc accumulation. (**A**) Haplotype heatmap surrounding significant SNPs on chromosome 1B; (**B**) Analysis of variance for haplotypes with different alleles and haplotype combinations in the two blocks; (**C**) Comparison between superior and inferior haplotypes based on phenotypic values collected from the four environments. Two symbols (* and ***) correspond to two *p*-values (0.05 and 0.001, respectively).

Among the 66 SNPs significantly associated with Zn grain in the KF location, 18 SNPs were located on the 1B chromosome. According to the genome-wide haplotype association analysis, these SNPs were clustered into two blocks, where block1 (*Hap1*) contained 4 SNPs with 2 haplotypes (*Hap1A* and *Hap1B*) and block2 (*Hap2*) contained 14 SNPs with also 2 possible haplotypes (*Hap2A* and *Hap2B*) (Figure 5A). Interestingly, when analyzing the effect of each haplotype on the phenotypic variation across the three environments, it appeared that variation in *Hap1* did not significantly affect Zn grain accumulation in any of the environments. Differently, variation in the *Hap2* was associated with different Zn grain levels in both the YY, SQ, and KF environment (Figure 5B). In this haplotype in fact was also located the SNP-based marker AX-111144645, which was the most significant SNP on chromosome 1B identified at the KF location (Table 2). When analyzing the combined effect of the *Hap1* and *Hap2* blocks the combination *Hap1A+Hap2A* had higher frequency (76.92%) compared to the haplotype *Hap1B+Hap2B* (23.08%), with this last combination being associated with higher Zn grain content in both the SQ, KF, and BLUP environment (Figure 5C).

### 2.5. Prediction of Candidate Genes

The recently annotated wheat genome reference sequence (IWGSC RefSeq v2.0) was employed to identify the candidate genes possibly associated with the seven major QTLs for Zn grain accumulation. The expression profiles of the candidate genes were obtained from the public database of Wheat Expression Browser (http://www.wheat-expression.com) and then, based on both the gene annotation information and the gene expression profiles, seven promising genes were selected as candidate genes relevant to Zn accumulation in wheat grains (Table 3). The two SNPs, which resulted to be significantly associated with grain Zn in all the four environments, coincided with the gene *TraesCS3D02G078500* on chromosome 3D and with the gene *TraesCS4A02G428500* on chromosome 4A. The first gene encoded for a *NAC domain-containing protein*, relevant to metal accumulation in wheat, whereas the second gene encoded for a *pentatricopeptide repeat-containing protein*. The SNP AX-112289745, located on chromosome 5B and identified in three different locations, was associated with the predicted gene *TraesCS5B02G069200*, encoding a protein in the family of the *serine/threonine-protein kinases*. Among the SNPs that were significantly associated with Zn accumulation in two environments, the SNP AX-110038787 on chromosome 1B resulted to be located in one of the exons of the predicted gene *TraesCS1B02G337400*, which encodes a *tetratricopeptide repeat (TPR)-like superfamily protein*. Differently, the SNP AX-110922471 located at chromosome 3B, was associated with the gene encoding a *CTP synthase* (*TraesCS3B02G252000*), involved in metal transport. The gene *TraesCS5A02G475200* that encodes for a *heavy metal transport/detoxification superfamily protein* associated with the homeostasis of Zn in plants, was located around 30kb away from the significant SNP marker on chromosome 5A (AX-110931014). Finally, the SNP AX-111012263 on chromosome 7A was linked with the predicted gene *TraesCS7A02G263700* encoding for a transcription factor. Notably, when analyzing the gene expression database, all the above-mentioned genes were expressed in wheat grains (Figure S2). A more detailed analysis of these candidate genes could provide useful information on Zn accumulation in wheat grains.

## 3. Discussion

Genetic biofortification is the most cost-effective and efficient approach to improve Zn grain content in wheat. However, to effectively improve this trait in a breeding program, it is of paramount importance to understand the genetics behind Zn grain accumulation. The GWAS analysis is an ideal approach to dissect the genetics of such a complex trait. For this reason, in the present study we conducted a GWAS analysis using a diverse panel of 207 individuals, which were genotyped through the 660K SNP array. From this analysis 125 marker-trait associations were identified, distributed in 29 non-redundant QTLs. Among them, 7 genomic regions were consistent across environments and associated with predicted genes possibly involved in the micronutrients uptake, translocation, and sequestration.

The primary consideration when performing a GWAS analysis is the identification of the best statistical model that allows the detection of the causal variant in the most efficient way (as low as possible false positives). It is important to consider this factor because different traits have different sensitivities to the model used for the GWAS analysis and have specific trait dependence. For example, Liu et al. [34] tested both the GLM and MLM models to perform a GWAS study for grain protein content and thousand kernel weight in wheat lines derived from *wild emmer*, and the MLM model resulted to be slightly better than GLM model for all traits. Differently, in the study performed by Liu et al. [35], FarmCPU was suggested to be the best choice for a GWAS analysis because of its higher statistical power compared to the GLM and MLM models. In the present study, after comparing both the GLM, MLM, and FarmCPU models, MLM appeared to be the best choice to perform a GWAS analysis on Zn grain accumulation. These results were in accordance with previous GWAS studies for Zn grain accumulation in wheat [29,30], where the association analysis was performed using the mixed linear model.

Slight differences were observed among the Zn concentrations in the grains of the association population planted in different locations, which may be attributed to varying environmental conditions of different locations (Figure 1A). The interaction between environments and genotypes has been reported in grains of other crops [36]. In this study, more significant SNPs were detected in KF (64 significant SNPs), while the number of significant SNPs from YY (12 significant SNPs) and SQ (10 significant SNPs) were obviously lower than KF. Moreover, compared with KF, more co-localization SNPs were detected between YY and SQ (Table S2). It is speculated that this may be resulted from the fact that the soil types of SQ and YY were more similar. Nevertheless, we identified several common genetic loci regulating Zn accumulation across all three environments (Table 2). Key genes underlying these loci are yet to be identified. These results implied that environmental characteristics including soil types should be taken into account in genetic analysis of zinc accumulation in wheat grains.

Many QTLs for Zn accumulation in grains have been detected by bi-parental linkage mapping [19,23,31,32,37], which allowed a comparison between known QTLs and loci identified in the present study. In the current study, 29 significant non-redundant loci were scattered across all wheat genome (except on chromosomes 2A, 4D, 5D, 7B, and 7D). Using a *durum wheat × wild emmer wheat* RIL population were identified stable QTL on chromosomes 5A, 6B and 7A, explaining 1.3% to 23.5% of the grain zinc variation [20]. These loci coincided with the loci identified in our study, on chromosomes 5A (AX-110931014, 650.24Mb), 6B (AX-109538092, 708.67Mb), and 7A (AX-111012263, 261.69Mb). Similarly, in a study conducted on a hexaploid wheat RIL, populations were identified five loci on chromosomes 1B, 2B, 3A, 3B, and 6A associated with Zn grain accumulation and explaining from 9% to 15% of the phenotypic variation [31]. In this study, we also identified 7 unique loci in 1B, 2B (2 loci), 3A, 3B (2 loci), and 6A, which could be associated with the QTL in the hexaploid bread wheat population [31]. However, like any other complex trait, Zn grain accumulation is controlled by a combination of multiple genetic loci and is also highly affected by the environment [38]. Therefore, the ideal targets would be the loci associated with Zn grain accumulation which can be stably identified in multiple environments. In the present study, the association analysis revealed that seven non-redundant genomic regions (on chromosomes 1B, 3B, 3D, 4A, 5A, 5B, and 7A) were identified in at least two environments, suggesting that these are stable QTLs, significantly associated with wheat grain Zn accumulation. Compared with previous research, a large proportion of these loci (except AX-94729264 on chromosome 3D) with relatively higher PVE values (11.97%-23.42%), were also detected in different genetic backgrounds. For example, the SNPs AX-110922471 (3B) and AX-112289745 (5B) were co-located with *QGZn.cimmyt-3B_1P2* and *QGZn.cimmyt-5B_P2* identified in the RIL population developed from the cross *SeriM82 × CWI76364* [39]. The two SNPs AX-110038787 and AX-108912427 on chromosomes 1B and 4A were mapped in genomic regions close to the *QGZn.sar_1BTSK* and *gwm397-gwm269* loci, respectively, the first identified in a RIL hexaploid wheat mapping population [25] and the second in a DH population [19]. Interestingly, among these genetic loci, the most significant and stable SNP (AX-94729264) identified on chromosome 3D could not be associated with any previously reported QTL for Zn grain content, indicating that, by using the population selected in the present study, we were able to find a novel stable QTL associated with accumulation of Zn in wheat grain. The above genetic detection results imply that zinc grain accumulation is controlled by many genetic loci distributed on the whole genome.

In the current study, seven significantly SNPs were significantly associated with wheat grains Zn concentration in at least two environments. These candidate regions together with genes previously shown to regulate metal homeostasis in plant, are considered promising genes for further validation in hexaploid wheat. Based on both the gene functional annotation and their relative expression level, seven genes were identified as potential candidate genes for Zn grain accumulation (Table 3). The most significant SNP AX-94729264 with highest average PVE (19.37%) on chromosome 3D was associated with a gene encoding a *NAC transcription factor*. This gene family contain a highly conserved DNA binding *NAC* domain in the N-terminal, which accelerates the senescence and also affects nutrient remobilization from leaves to developing wheat grains during grain filling period [40]. The second significant SNP AX-108912427 on chromosome 4A with PVE average value equaling 18.58% was associated with a gene encoding for a *V-type proton ATPase*. This protein has a typical ATP synthesis activity, with an important function for membrane trafficking, and serves as generator of an electrochemical gradient driving the accumulation of nutrients within plant cells vacuole, which may be related to cells ion homeostasis [41,42]. The marker AX-110038787 on chromosome 1B was identified within the exon of a gene encoding a protein that contains *tetratricopeptide (TPR) repeats*, which may mediate protein-protein interactions and chaperone activity thus affecting the activity of osmotic stress responses and metal ion binding [43]. Similarly, the marker AX-112289745 on chromosome 5B was also identified in the exon of a predicted gene which, in this case, encoded for a *serine/threonine-protein kinase*, which serves as the mediator for the regulation of voltage-dependent ion channels according to protein phosphatases [44]. On chromosome 3B, one locus (containing one SNPs AX-110922471) was associated with the predicted gene *TraesCS3B02G252000* encoding a *CTP synthase*, which using L-glutamine as the nitrogen source catalyzes CTP from UTP and is involved in the conversion between glutamine and CTP. However, the glutamine is an important N-source in organisms, and previous research have shown that sufficiently high nitrogen utilization could facilitate Zn from vegetative tissues to grain, while affecting the Zn concentration in grains [45,46]. The remaining SNPs AX-111012263 on chromosome 7A and AX-110931014 on chromosome 5A were associated with candidate genes *TraesCS7A02G263700* and *TraesCS5A02G475200*, respectively, the first encoding for a *basic helix-loop-helix transcription factor (bHLH)* and the second for a *heavy metal transport/detoxification superfamily protein*. Overexpressing *bHLH* transcription factor will increased tolerance for Zn toxicity [47]. In addition, the *heavy metal transport/detoxification superfamily protein* might be involved in altered zinc homeostasis in wheat. Interestingly, all the above-mentioned candidate genes exhibited high expression levels in wheat grains (Figure S2). Specifically, the candidate gene *TraesCS3D02G078500* associated with the most significant SNP, was only expressed in wheat grains, suggesting its importance in the grain nutrient accumulation.

Grain yield is the major target of any breeding program. However, higher yield (and greater grain size) is often associated with considerable decreases in the concentrations of Zn in wheat grains, because of the so-called “dilution effect” [33,48,49]. For this reason, it is difficult to screen zinc-rich wheat varieties at high yield levels. Nevertheless, using molecular marker-assisted (MAS) breeding, pyramiding favorable alleles associated with micronutrient accumulation could significantly enhance the concentrations of necessary micronutrient elements in wheat grains, including Zn [15,22,50]. Therefore, we compared phenotypic values collected from four environments (KF, YY, SQ, and BLUP) and found that genotypes harboring the favorable alleles at the seven non-redundant loci exhibited significantly higher Zn grain content values. These results would indicate that multi-loci pyramid breeding could be ideal to improve Zn-grain content. Furthermore, compared the Zn-grain content of cultivars with superior haplotype and inferior haplotype on chromosome 1B, the results of the *Hap1B+Hap2B*’s phenotypic performance was better than *Hap1A+Hap2A*, and the difference reached a significant level. It suggested that the superior haplotype (*Hap1B+Hap2B*) with great positive effect for Zn accumulation in wheat grain. The above results imply that grain Zn accumulation is largely controlled by multiple genetic loci with mainly additive effects.

## 4. Materials and Methods

### 4.1. Plant Materials

Based on the pedigree, physiological characteristics, and adaptive fields, a representative sample of 207 wheat cultivars was used in the association population. The selected cultivars originated from the Yellow and Huai Valley and Southwestern Wheat Region of China, including Henan, Hebei, Shaanxi, Shanxi, Shandong, Jiangsu, Sichuan, and Yunnan provinces. The association population was planted at Yuanyang (YY, E113°97′, N35°5′, 78 m above mean sea level), Kaifeng (KF, E114°30′, N34°80′, 72.5 m above mean sea level) and Shangqiu (SQ, E115°65′, N34°45′, 50.1 m above mean sea level) of the Huang-Huai Plain, China, during October 2016 to June 2017. The three sites are located in northern (YY) and east (KF and SQ) Henan Province, with an average annual temperature of 14.3 °C, 15.0 °C and 14.2 °C, respectively. The average annual rainfalls of YY, KF and SQ are 556 mm, 650 mm and 623 mm, separately. The major soil type of the three locations are fluvo-aquic soil with different sand content (YY, sand 27%; KF, sand 55%; SQ, sand 24%; an Inceptisol in the USDA soil taxonomy system), developed from alluvial sediments of the Yellow River [51]. In each cropping location, the field experiment was organized in a randomized complete block design. Each plot contained five rows (2 m long each) at a density of 10 cm space between plants and 23 cm space between rows. All wheat accessions were grown in each location using standard agronomic practices.

### 4.2. Determination of the Zn Concentration in Wheat Grains

For three different locations, the wheat grains of 207 cultivars were harvested and analyzed at each location to minimize variation across different environments. The association population was harvested at physiological maturity (8–10% moisture content) to determine the accumulation and distribution of Zn in wheat grains. Approximately 20g grain samples of each entry were threshed by hand and carefully removed the broken grains and foreign materials. The samples were stored in glassine bags to ensure that no contact with any metal containers. The Zn grain content was estimated following the protocol of Zhang et al. [52]. Briefly, wheat grains were washed quickly with 0.1 N HCl to remove any surface contaminants and dried in hot air oven at 80 °C. The dry grains were first ground into a fine powder using grinding miller. Then the powder samples were digested with 5mL mixture of concentrated nitric acid and perchloric acid (HNO_3_/HClO_4_, 80/20, *v*/*v*, analytical reagent grade, Merck, Darmstadt, Germany) through a heating block (AIM500 Digestion System, A.I. Scientific, Canberra, Australia). The Zn concentration was determined using atomic fluorescence spectrometry (AFS-3000, Beijing Haiguang Analytical Instrument Co., Beijing, China). Each sample from the three locations was analyzed three times to generate technical replicates and the average values among three technical replicates were used for further analysis (Table S1).

### 4.3. Statistical Analysis

A frequency map was made using R software version 3.5.3 (R Development Core Team, 2019). Descriptive statistics (such as mean, range, and the normal distribution description parameters “skewness” and “kurtosis”) for all of the phenotypic data from each location, was conducted in R software with the “psych” package. Best linear unbiased predictors (BLUPs) values for each cultivar across the three locations were obtained by fitting the mixed linear model in R package “lem4” and were computed according to the formula: Y = (1|Line) + (1|Loc) + (1|Rep%in%Line: Loc) + (1|Line: Loc). In the above formula, Y represents the trait value, the values in the parenthesis indicate the random effects, “:” indicates interactions, and “1|” means groups. “Line” and “Loc” indicate all the testcrosses and environments, respectively. Additionally, “Rep” refers to the replications in one location. Then, the BLUP values were combined to reduce the prediction bias caused by the unbalanced data. Finally, the BLUP values for the Zn contents of each accession across three environments were used as the input data for the further association mapping analysis.

### 4.4. SNP Genotyping and Filtering

Total DNA was extracted from young leaves tissue of the accessions using a modified CTAB procedure previously reported [53]. The DNA quality, purity, and concentration for each sample was verified by gel-electrophoresis and spectrophotometer (NanoDropTM One, Thermo Fisher Scientific, Wilmington, DE, USA). The panel consisting of 207 common wheat cultivars was genotyped using the Wheat Breeders 660 K Axiom^®^ array according to the Axiom 2.0 Assay Manual Workflow protocol [54]. After genotyping, the raw data was filtered as suggested in the Plink v1.9 software using the following settings: ‘--maf 0.05 --geno 0.1′, that means a minor allele frequency (MAF) > 0.05 and the genotype missing data less than 10% [55]. In total 244,508 SNP markers were used for the GWAS and following analysis.

### 4.5. GWAS

The population structure was calculated using the Structure software version2.3.4 (Pritchard Lab, Stanford University, San Francisco, CA, USA) with admixture model. An admixture model with ten replicates for each genetic group (*K* = 1–10). A burn-in of 1000 iterations followed by 1000 MCMC (Markov Chain Monte Carlo) replicates was used to estimate the number of subpopulations. The optimal K value was determined using the method of ∆K [56]. The variance-covariance kinship matrix was calculated using the VanRaden method to determine relative kinship among the sampled individuals [57]. GWAS was implemented for wheat grains Zn contents at YY, KF, SQ, and the combination of three locations. In order to select the optimal GWAS model, the GLM (only account for population structure), MLM (account for population structure and relative kinship), and FarmCPU (account for fixed and random effect model) models were adopted with GAPIT package in R software [58] to analyze the association between genotype and phenotype. Because several SNPs should be in linkage disequilibrium, the GEC software tool was used to calculate the effective number of independent markers [59]. The significant *p*-values was then adjusted accordingly to 1.0 × 10^−4^ in order to control the genetic type I error rate for this population.

### 4.6. Haplotype Block and Superior Allele Estimation

Whole genome haplotype blocks of the natural population were estimated using Plink version1.9 with following settings: ‘--blocks no-pheno-req --blocks-max-kb 500′. The haplotype blocks with positive effects leading to higher Zn accumulation were considered as “superior haplotypes”. In contrast, the haplotype blocks with negative effects leading to lower Zn accumulation in wheat grains were considered as “inferior haplotypes”. Each locus comprises two alleles based on SNP marker.

### 4.7. Candidate Gene Identification

Based on the reported common wheat reference genome sequence, the high confidence wheat gene list was downloaded from the International Wheat Genome Sequence Consortium (IWGSC) website (https://wheat-urgi.versailles.inra.fr/). After GWAS, the DNA sequence flanking the SNP markers significantly associated with Zn accumulation, were analyzed against the “IWGSC RefSeq v1.0” using Basic Local Alignment Search Tool (BLAST) in NCBI databases with default parameters (https://blast.ncbi.nlm.nih.gov/Blast.cgi). The physical positions of SNP markers were then obtained from the public database of JBrowse (http://202.194.139.32/jbrowse/?data=Chinese_Spring). Candidate genes transcripts and their corresponding annotation information were obtained from the website of IWGSC (https://urgi.versailles.inra.fr/jbrowseiwgsc/gmod_jbrowse/IWGSC_RefSeq_v1.0). For the loci without appropriate candidates, the gene nearest to the peak SNP was assigned. All of the potential candidate genes and their corresponding annotations within all detected loci were listed in Table 3.

## 5. Conclusions

The results obtained from the GWAS analysis conducted in the present study provide an overview on the genomic regions involved in Zn grain accumulation in a wheat diversity panel. A total of 29 loci were significantly associated with Zn accumulation in wheat, and seven loci were associated with Zn grain accumulation in multiple environments. The associated PVE values ranged from 11.97% to 24.77% in Yellow and Huai wheat region. Putative candidate genes associated with the most significant QTL were identified and could be possible targets to elucidate mechanisms of wheat grains Zn accumulation in the future. The SNPs most highly associated with Zn grain accumulation could be converted into molecular markers to improve the efficiency of breeding zinc-rich wheat varieties. Overall, this study improves the understanding of the genetic architecture behind Zn accumulation in wheat grains, thus facilitating the future improvement of Zn grain concentrations in wheat breeding programs.

## Figures and Tables

**Table 1:** Descriptive statistics for the zinc contents in the association population.

Location	Trait	Mean ± SD ^1^ (mg/kg)	Range (mg/kg)	Kurt ^2^	Skew ^3^
Yuanyang (YY)	Zn content	65.63 ± 18.57	31.87–116.47	−0.61	0.31
Kaifeng (KF)	Zn content	46.45 ± 17.98	14.24–99.00	0.14	0.53
Shangqiu (SQ)	Zn content	68.63 ± 18.23	25.83–128.22	0.01	0.08
BLUP	Zn content	60.24 ± 2.63	54.65–69.65	0.20	0.35

^1^ SD, Standard Deviation; ^2^ Kurt, kurtosis, which is a measure of the ‘tailedness’ of the probability distribution of a real-valued random variable; ^3^ Skew, skewness, which is a measure of the asymmetry of the probability distribution of a real-valued random variable about its mean.

**Table 2:** List of significant loci and their detailed information for Zn content identified by genome-wide association study (GWAS).

ID ^1^	Chromosome	Interval Range (bp)	No. of SNPs	Location	Peak SNP ^2^	Position (bp) ^3^	*p* Value ^4^	*R*^2^ (%) ^5^
1	1A	574,477,246–574,479,186	3	KF	AX-110606195	574,477,470	3.27 × 10^−5^	10.26
2	1A	592,315,138	1	YY	AX-108995328	592,315,138	3.55 × 10^−5^	12.27
3	1B	564,909,314	1	YY	AX-110038787	564,909,314	1.03 × 10^−5^	13.53
				BLUP	AX-110038787	564,909,314	4.33 × 10^−5^	13.78
4	1B	665,798,565–668,100,871	18	KF	AX-111144645	668,002,032	2.69 × 10^−7^	15.44
5	1D	16,132,987	1	SQ	AX-110529533	16,132,987	1.46 × 10^−5^	12.64
6	1D	478,184,386	1	KF	AX-110828223	478,184,386	8.47 × 10^−5^	9.27
7	2B	154,930,484	1	YY	AX-110620516	154,930,484	8.62 × 10^−5^	11.37
8	2B	787,099,530	1	KF	AX-109490599	787,099,530	5.82 × 10^−5^	9.66
9	2D	1,638,170	1	SQ	AX-94598102	1,638,170	9.19 × 10^−5^	10.70
10	2D	519,133,490–650,654,168	3	YY	AX-94583825	582,025,967	5.40 × 10^−5^	11.84
				KF	AX-94466886	650,654,168	2.75 × 10^−5^	10.44
11	3A	219,103,987–260,492,627	5	KF	AX-108914831	235,844,032	4.80 × 10^−5^	9.86
12	3B	376,625,452	1	SQ	AX-110922471	376,625,452	2.09 × 10^−6^	14.75
				YY	AX-110922471	376,625,452	5.69 × 10^−8^	19.07
13	3B	779,542,533	1	YY	AX-94567805	779,542,533	7.58 × 10^−5^	11.50
14	3D	40,526,440	1	KF	AX-94729264	40,526,440	1.24 × 10^−6^	13.76
				SQ	AX-94729264	40,526,440	4.45 × 10^−10^	24.55
				YY	AX-94729264	40,526,440	3.69 × 10^−10^	24.77
				BLUP	AX-94729264	40,526,440	2.32 × 10^−5^	14.40
15	3D	515,115,709–519,527,578	3	BLUP	AX-111858412	515,115,709	3.18 × 10^−5^	14.08
16	4A	669,454,046–699,571,654	2	SQ	AX-108851891	669,454,046	5.27 × 10^−5^	11.28
				KF	AX-108912427	699,571,654	4.47 × 10^−7^	14.87
				SQ	AX-108912427	699,571,654	1.14 × 10^−9^	23.42
				YY	AX-108912427	699,571,654	1.92 × 10^−9^	22.87
				BLUP	AX-108912427	699,571,654	8.02 × 10^−5^	13.17
17	4B	13,996,819	1	SQ	AX-89748062	13,996,819	1.81 × 10^−5^	12.42
18	5A	42,281,607	1	BLUP	AX-94932868	42,281,607	6.31 × 10^−5^	13.41
19	5A	549,409,584–552,208,450	23	KF	AX-111463331	549,576,304	4.61 × 10^−6^	12.33
20	5A	650,240,330	1	SQ	AX-110931014	650,240,330	9.82 × 10^−7^	15.59
				YY	AX-110931014	650,240,330	1.08 × 10^−6^	15.89
				BLUP	AX-110931014	650,240,330	3.39 × 10^−5^	14.02
21	5B	57,158,679–57,493,343	3	BLUP	AX-110398218	57,493,343	3.92 × 10^−5^	13.87
22	5B	78,708,064	1	KF	AX-112289745	78,708,064	2.47 × 10^−6^	13.00
				SQ	AX-112289745	78,708,064	1.55 × 10^−5^	12.58
				YY	AX-112289745	78,708,064	3.29 × 10^−7^	17.16
23	5B	407,053,365–412,175,187	41	BLUP	AX-86176241	411,929,890	3.15 × 10^−5^	14.09
24	5B	693,033,900	1	KF	AX-110443373	693,033,900	8.15 × 10^−6^	11.72
25	6A	613,482,310	1	KF	AX-94961930	613,482,310	1.72 × 10^−5^	10.93
26	6B	462,555,585	1	YY	AX-111084964	462,555,585	5.22 × 10^−5^	11.88
27	6B	708,670,301	5	KF	AX-109538092	708,670,301	2.09 × 10^−5^	10.73
28	6D	464,120,129	1	KF	AX-110536000	464,120,129	5.33 × 10^−5^	9.75
29	7A	261,687,749	1	SQ	AX-111012263	261,687,749	8.04 × 10^−6^	13.28
				YY	AX-111012263	261,687,749	3.21 × 10^−6^	14.74

^1^ The ID of the loci identified in GWAS; ^2^ Most significant SNP on each locus; ^3^ The peak SNPs physical position according to IWGSC RefSeq v2.0 of the bread wheat reference; ^4^ *p* value of the corresponding trait calculated by MLM model; ^5^ The phenotypic variance explained by the corresponding locus.

**Table 3:** Significant SNP identified in current and previous study.

ID ^1^	Chromosome	Identified Loci in Current Study	Position (bp) ^2^	Near Locus Previously Reported in the Same Chromosome	Candidate Genes (Closest/Nearby)	Annotation
1	1B	AX-110038787	564,909,314	*QGZn.sar_1BTSK* [19]	TraesCS1B02G337400	Tetratricopeptide repeat (TPR)-like superfamily protein
2	3B	AX-110922471	376,625,452	*QGZn.cimmyt-3B_1P2* [31]	TraesCS3B02G252000	CTP synthase
3	3D	AX-94729264	40,526,440	-- ^3^	TraesCS3D02G078500	NAC domain-containing protein
4	4A	AX-108912427	669,454,046	*QGZn.iari-4A* [32]	TraesCS4A02G428900	V-type proton ATPase
				*gwm397-gwm269* [19]		
5	5A	AX-110931014	650,240,330	*Xgwm291-Xgwm410* [33]	TraesCS5A02G475200	Heavy metal transport/detoxification superfamily protein
				*Xbarc223.1-Xswes157* [22]		
6	5B	AX-112289745	78,708,064	*QGZn.cimmyt-5B_P2* [31]	TraesCS5B02G069200	Serine/threonine-protein kinase/Kinase family protein
7	7A	AX-111012263	261,687,749	*GZn.pau-7A* [21]	TraesCS7A02G263700	Basic helix-loop-helix transcription factor

^1^ The repetitive significantly SNP loci; ^2^ Physical position of the SNP as reported in the IWGSC Chinese Spring reference genome RefSeq v2.0; ^3^ “--” indicated that there is no co-localized QTLs at this locus.

**Table 4:** Analysis of variance (ANOVA) for individuals harboring the superior and inferior alleles for the seven most significant and consistent SNP in different environments.

SNP_id	Chr.	Allele Type		Phenotype Value		Allele Number		Allele Percentage (%)		*p*-Value			
		Superior	Inferior	Superior	Inferior	Superior	Inferior	Superior	Inferior	Zn_YY	Zn_SQ	Zn_KF	Zn_BLUP
AX-110038787	1B	CC	GC	60.79	58.78	148	55	72.91	27.09	2.90 × 10^−7^	9.36 × 10^−4^	4.74 × 10^−1^	7.42 × 10^−7^
AX-110922471	3B	AA	GG	60.77	59.16	53	86	38.13	61.87	2.93 × 10^−6^	7.28 × 10^−4^	2.87 × 10^−1^	6.39 × 10^−4^
AX-94729264	3D	CT	CC	61.27	59.43	90	113	44.33	55.67	7.11 × 10^−14^	4.09 × 10^−14^	6.03 × 10^−7^	3.31 × 10^−7^
AX-108912427	4A	AG	GG	61.24	59.43	90	111	44.78	55.22	1.33 × 10^−13^	1.33 × 10^−13^	7.85 × 10^−7^	6.32 × 10^−7^
AX-110931014	5A	AA	CC	60.90	59.20	17	94	15.32	84.68	1.68 × 10^−2^	5.89 × 10^−2^	4.80 × 10^−1^	1.81 × 10^−2^
AX-112289745	5B	AG	GG	60.98	59.49	92	95	49.20	50.80	9.72 × 10^−11^	9.95 × 10^−8^	1.35 × 10^−5^	1.22 × 10^−4^
AX-111012263	7A	GG	AG	60.96	59.22	94	86	52.22	47.78	2.23 × 10^−10^	4.53 × 10^−10^	5.08 × 10^−5^	2.71 × 10^−6^

## Supplementary Materials

Supplementary materials can be found at https://www.mdpi.com/1422-0067/21/6/1928/s1.

## Abbreviations

ANOVA	Analysis of variance
BLUP	Best linear unbiased predictors
FarmCPU	Fixed and random model Circulating Probability Unification
GLM	General linear model
GWAS	Genome-wide association studies
IWGSC	International Wheat Genome Sequence Consortium
MLM	Mixed linear model
PVE	Phenotypic variation explained
QQ	Quantile–quantile
QTL	Quantitative trait loci
SNP	Single nucleotide polymorphism
Zn	Zinc
