# Supplementary material for: Identification of Novel Genomic Regions and Superior Alleles Associated with Zn Accumulation in Wheat Using a Genome-Wide Association Analysis Method
Source: Int J Mol Sci. 2020 Mar 11;21(6):1928. doi: 10.3390/ijms21061928 (PMC7139793; doi:10.3390/ijms21061928)
Supplement: Supplementary file 1 [file ijms-21-01928-s001.pdf]

Article

Identification of novel genomic regions and superior alleles associated with Zn accumulation in wheat using a genome-wide association analysis method

Zhengfu Zhou [1,†], Xia Shi [1,†], Ganqing Zhao [2], Maomao Qin [1], Maria Itria Ibba [3], Yahuan Wang [1], Wenxu Li [1], Pan Yang [1], Zhengqing Wu [1,4], Zhensheng Lei [1,2,4,*], Jiansheng Wang [2,*]

[1] Wheat Research Institute, Henan Academy of Agricultural Sciences, Zhengzhou 450002, Henan, China; zhouzf215@163.com (Z.Z.); mrshi0614@126.com (X.S.); qmm1988630@126.com (M.Q.); wyh.best@163.com (Y.W.); tinbingye@163.com (W.L.); ypang0916@163.com (P.Y.), wzhqfy@163.com (Z.W)

[2] College of Chemistry and Environment Engineering, Pingdingshan University, Pingdingshan 467000, Henan, China; zgq1118@163.com (G.Z.)

[3] Global Wheat Program, International Maize and Wheat Improvement Center (CIMMYT), Mexico, D.F. 06600, Mexico; M.IBBA@cgiar.org (M.I.I.)

[4] College of Life Sciences, Zhengzhou University, Zhengzhou, Henan 450001, China

* Correspondence: zhenshenglei@126.com (Z.L.); wjsheng1998@163.com (J.W.)

Tel: +86-371-6572-4084 (Z.L.); +86-158-3759-0332 (J.W.)

[†] These authors contributed equally to this work.

SUPPLEMENTAL INFORMATION

This file includes Supplemental Tables1-2.

Supplemental Figure S1-2 are separate figure files and are available at International Journal of Molecular Sciences Online.

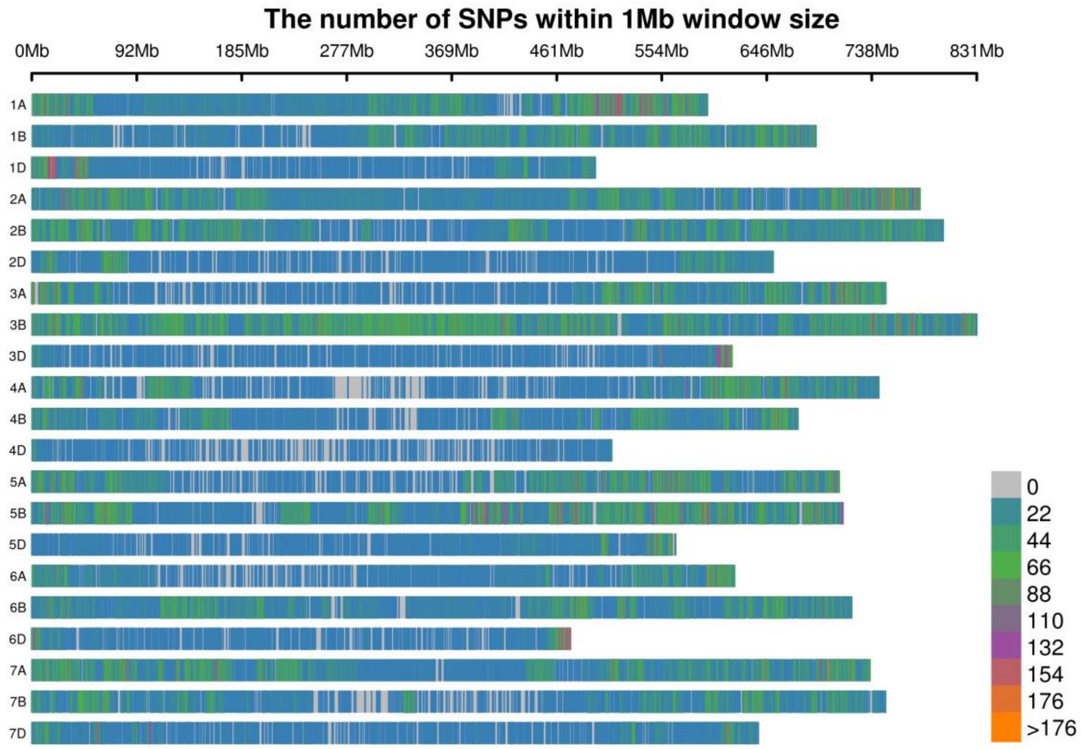

Figure S1. Single nucleotide polymorphism (SNP) density (number of SNPs within 1Mb window size) of 207 bread wheat lines analyzed with the 660K SNP arrays.

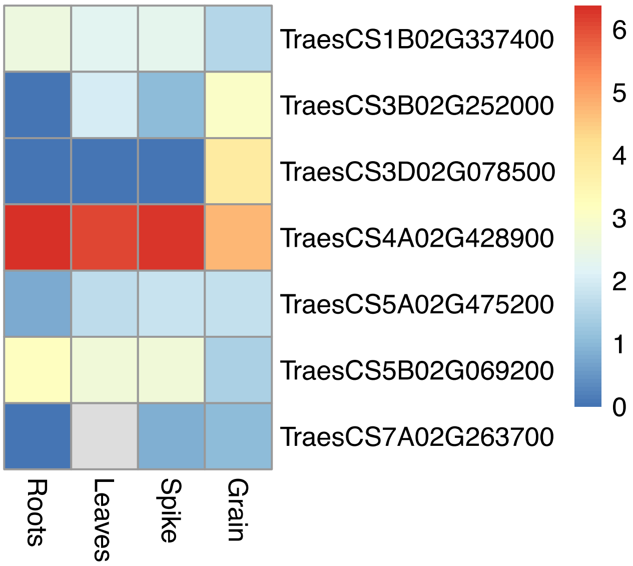

Figure S2. Expression level of seven promising candidate genes in different wheat tissues. The heat map was plotted using the transcripts per kilobase million (TPM) values from the gene expression profile public database.

Table S1. Average phenotypic values of Zn accumulation in 207 wheat accessions across from each environments and BLUP.

Accession ID.	Cultivar name	YY	SQ	KF	BLUP
L001	Changwu 135	51.65	104.96	67.96	67.63
L002	Shan 229	81.45	158.11	57.78	77.31
L003	Xiaoyan 6	77.32	193.21	66.06	82.53
L004	Lantian 10	87.00	167.05	62.91	79.92
L005	Luohan 3	80.30	313.40	62.25	98.41
L006	Jing 411	44.12	182.94	50.74	74.71
L007	Ningdong 1	54.80	162.33	83.61	77.76
L008	Mianyang 11	75.25	85.84	56.54	66.71
L009	Taishan 5	122.75	168.87	74.83	86.50
L010	Xinmai 13	56.04	128.22	65.73	71.01
L011	Xinmai 18	85.10	168.62	57.95	79.22
L012	Zhoumai 16	41.64	143.54	27.81	66.09
L013	Yuanfeng 139	91.06	89.40	21.85	64.67
L014	Fengchan 3	92.96	87.58	20.03	64.44
L015	Zhongyu 8	72.35	94.70	24.67	63.26
L016	Bainong 160	99.83	69.78	15.73	62.41
L017	Luomai 21	67.71	84.60	21.70	60.91
L018	Yanzhan 4110	90.98	110.68	22.69	67.60
L019	Gan 6172	102.81	92.22	20.06	66.37
L020	Huaimai 21	91.39	105.38	19.56	66.53

L021	Mianyang 26	98.59	86.50	23.34	65.48
L022	Lunxuan 715	103.14	76.82	14.24	63.59
L023	Youpi 1	64.01	75.33	26.82	59.86
L024	Xifeng 9	71.52	92.96	16.72	61.86
L025	Nongda 198	67.13	78.97	24.92	60.51
L026	Kedong 81	59.60	91.88	47.18	64.18
L027	Linfeng 10	84.60	122.35	33.69	69.77
L028	Fengkang 5	83.61	96.35	37.58	66.70
L029	Changfeng 1	74.00	76.32	23.18	60.84
L030	Jingdong 1	82.61	79.30	23.34	62.40
L031	Jinmai 21	77.32	30.30	18.86	54.58
L032	Jimai 23	88.24	83.44	20.36	63.30
L033	Hanxuan 10	76.65	105.21	19.26	64.51
L034	Xianshixinmai	58.11	60.35	23.01	56.58
L035	Duckbill wheat	75.58	63.16	16.72	58.44
L036	Bima 1	71.02	72.18	25.25	60.17
L037	Aimengniu	63.14	70.25	26.74	59.06
L038	Wenmai 6	78.89	73.10	20.03	60.64
L039	Zhameng wheat	68.21	71.43	25.16	59.68
L040	Dayuhua	67.13	70.80	18.94	58.63
L041	Chushanbao	67.98	71.21	23.18	59.36
L042	Dalibanmang	86.92	76.49	22.93	62.55
L043	Baizhameng	95.69	75.62	15.73	62.64

L044	Ganmai 8	58.81	69.46	24.50	58.08
L045	Yunmai 34	86.01	84.45	41.47	65.95
L046	Wuyi wheat	59.10	69.33	26.74	58.40
L047	Nanda 2419	77.98	76.22	32.12	62.54
L048	Nonglin 10	108.77	86.61	23.67	66.90
L049	Hanyang wheat	90.73	91.49	24.67	65.28
L050	Lovelin 10	50.33	68.27	47.85	59.90
L051	Aifeng 3	69.37	72.65	32.70	61.00
L052	Yunhan 618	71.69	72.92	28.72	60.82
L053	Yannong 21	71.69	74.07	35.26	61.84
L054	Jinmai 47	73.59	72.72	24.25	60.45
L055	Changwu 58	86.42	82.03	35.76	64.92
L056	Zhenghan 1	71.36	73.09	30.46	61.03
L057	Chang 6878	74.50	74.27	30.30	61.58
L058	Luohan 1	70.03	71.64	23.01	59.66
L059	Luohan 6	93.54	88.53	39.24	67.20
L060	Luohan 11	89.48	84.04	36.09	65.64
L061	Puxing 5	78.64	83.26	51.99	66.21
L062	Ruiquan 24	67.38	72.80	41.22	61.89
L063	Dehongfu 2	85.43	91.21	56.12	68.72
L064	Zhongmai 895	93.13	96.72	52.81	70.04
L065	Huaimai 35	75.00	79.62	49.83	64.95
L066	Longping 518	82.12	79.21	34.85	63.85

L067	Huaimai 30	74.34	79.59	51.49	65.08
L068	Zhongyuan 6	71.02	75.24	43.21	62.96
L069	Pingan 8	84.60	83.89	42.38	65.81
L070	Bainong 207	79.30	82.94	49.67	65.95
L071	Cun 1	76.74	77.55	38.82	63.45
L072	Zhoumai 26	75.66	64.73	41.64	61.97
L073	Luomai 18	79.88	65.03	59.93	65.01
L074	Baofeng 10-82	69.87	66.14	38.41	60.96
L075	Su 553	98.84	76.65	41.47	66.62
L076	09N37	103.72	68.21	44.04	66.49
L077	Guinong 17	93.04	74.09	41.89	65.56
L078	Hengguan 35	71.85	58.47	40.40	60.47
L079	Yumai 18	65.48	54.47	45.53	59.77
L080	Yumai 13	83.44	71.44	43.62	64.16
L081	Yumai 47	116.47	77.98	47.85	69.99
L082	Zhengmai 004	68.87	82.20	53.56	64.98
L083	Yumai 50	60.59	71.69	89.40	67.25
L084	Taikong 6	43.71	58.66	46.19	57.52
L085	Huapei 5	91.72	77.40	51.57	67.11
L086	Hua 9987	55.13	87.50	60.10	64.73
L087	Yujiao 5	33.28	61.26	39.15	55.54
L088	Yunong 416	51.99	72.10	40.06	59.59
L089	Bainong 64	77.65	70.36	46.85	63.68

L090	Zhoumai 9	55.13	80.63	46.85	62.05
L091	Zhoumai 13	106.78	88.74	42.80	69.46
L092	Zhoumai 8425B	86.26	71.52	47.02	65.00
L093	Kaimai 21	55.38	77.89	45.53	61.54
L094	Luozhen 1	50.33	91.72	73.42	66.42
L095	Neixiang 188	73.51	69.04	61.59	64.91
L096	Aiyou 26-2	68.79	64.40	56.54	63.00
L097	Changgeheimai	47.68	87.41	65.73	64.47
L098	Lvmai 1	59.10	86.75	51.65	64.03
L099	Subeimai 1	56.12	61.09	34.02	57.88
L100	Jinan 17	47.18	77.98	49.50	60.99
L101	Jimai 20	49.58	79.96	37.91	60.03
L102	Shan 225	41.80	84.60	47.27	60.86
L103	Xinong 979	54.97	78.97	51.32	62.40
L104	Baxter	58.44	88.57	49.01	63.83
L105	CD87	65.06	92.55	55.13	66.06
L106	Kukri	49.01	83.77	59.52	63.34
L107	Faguomai	50.00	88.82	42.88	61.93
L108	Belero	61.42	56.24	58.94	61.25
L109	Fundulea 900	54.80	53.97	70.36	61.59
L110	Tincurrin	41.89	31.61	64.73	56.15
L111	H149	91.64	53.53	63.08	65.46
L112	Fa B08	44.87	41.36	62.17	57.50

L113	Fa B20	55.88	43.10	52.15	57.86
L114	Bainong 416	55.05	40.40	67.55	59.44
L115	Luo 31	50.66	42.43	69.53	59.39
L116	Taihemai 1	41.14	33.67	61.42	55.88
L117	Xunmai 35	39.90	25.98	52.98	53.57
L118	Luomai 23	34.27	25.83	66.55	54.61
L119	Jiaomai 266	38.57	49.01	57.45	57.05
L120	Wennong 14	36.67	43.02	67.55	57.34
L121	Jimai 22	38.41	39.97	74.91	58.15
L122	Ningmai 9	35.59	48.84	38.08	54.06
L123	10EW28	37.58	51.77	31.62	53.85
L124	10EW137	40.81	59.02	35.43	55.75
L125	Zhou 18	61.09	52.95	51.99	59.84
L126	04Z38	45.28	57.25	43.71	57.21
L127	Yumai 34-9901	46.52	45.50	35.10	54.67
L128	Zheng 9023-8	42.47	53.14	38.74	55.63
L129	Zheng 103	42.47	50.96	46.69	56.40
L130	Calingiri	31.87	49.42	26.65	52.12
L131	Zhengmai 366	44.70	59.63	40.06	56.97
L132	Zhengmai 7698	52.48	44.01	44.37	56.50
L133	Zhoumai 22	50.11	51.90	40.73	56.75
L134	Zhoumai 27	47.06	66.89	39.24	58.14
L135	Zhoumai 30	52.48	67.90	43.62	59.57

L136	Zhoumai 32	52.02	79.70	31.79	59.51
L137	Liangxing 66	40.93	67.65	39.32	57.43
L138	Emai 25	47.10	64.67	38.91	57.80
L139	Ningchun5	32.19	57.57	34.85	54.33
L140	Shannong 33	48.09	50.66	43.29	56.65
L141	Zhongmai 175	49.20	43.29	20.94	52.85
L142	Gaocheng 8901	36.07	42.03	38.99	53.33
L143	Shannongtedali 1	36.42	68.14	34.27	56.23
L144	Changmai 9	39.91	48.90	43.54	55.36
L145	Yangfumai 2	39.08	41.19	42.13	54.04
L146	Wanmai 53	37.01	48.76	34.44	53.75
L147	Fanmai 8	35.92	52.02	30.46	53.51
L148	Yunmai 51	66.22	40.94	52.15	58.95
L149	Yunmai 53	40.06	64.34	44.54	57.57
L150	Yunmai 47	56.79	41.72	38.24	55.95
L151	Xinong 928	53.56	60.15	32.45	57.20
L152	Xinmai 26	59.60	41.72	32.28	55.53
L153	Wanmai 47	57.45	60.13	33.19	57.81
L154	Yangmai 13	55.79	44.21	35.59	55.80
L155	Gaoyou 2018	52.62	57.55	67.13	61.34
L156	Gaoyou 9415	62.70	72.01	62.08	63.94
L157	Shiluan 02-1	58.44	53.48	58.11	60.38
L158	Zheng 1005	55.63	77.51	65.81	64.22

L159	Zheng 1105	58.19	61.53	61.01	61.80
L160	Zheng 1118	61.42	42.10	53.31	58.62
L161	Zheng 1289	58.77	53.07	26.92	56.22
L162	Zheng 3093	83.94	41.72	80.30	65.16
L163	Sanyuehuang	64.57	29.18	98.18	63.29
L164	Wuhuatou	66.64	89.57	92.71	70.87
L165	Tutoumai	82.94	53.56	111.92	70.80
L166	Baisuibai	78.31	88.48	92.38	72.23
L167	Qumangmai	114.15	320.69	105.46	109.63
L168	Zhengzhou 6	101.98	78.47	76.16	71.89
L169	Zhengyin 1	73.34	91.22	79.80	70.26
L170	Shengxuan 7	80.79	86.83	94.04	72.56
L171	Shuangfengshou	53.48	76.32	53.64	62.16
L172	Zhengzhou 7	77.81	52.65	49.17	61.65
L173	Wanchangsui	51.32	66.14	35.02	58.04
L174	Yuanzhu	72.10	48.09	41.39	59.25
L175	Longfumai 4	106.95	97.10	99.00	78.07
L176	Yizheng 8165	84.77	81.54	67.71	68.89
L177	Taihan 2	98.01	65.97	54.47	66.82
L178	Neixiang 182	69.53	88.66	46.03	64.92
L179	Chuanmai 50	70.03	36.75	44.29	57.85
L180	Chuanmai 46	65.48	57.45	60.79	62.20
L181	Chuanmai 44	70.36	41.14	61.59	60.78

L182	Chuanmai 107	75.99	70.69	44.95	63.25
L183	Jinyang 35	96.52	95.78	89.32	75.22
L184	Zhengmai 101	54.39	62.25	48.09	59.67
L185	Zhengmai 518	53.39	55.13	49.50	58.78
L186	AK 58	61.09	66.89	69.45	64.02
L187	Shannong 22	58.28	64.07	52.48	61.01
L188	Nongda 211	61.26	68.29	91.88	67.21
L189	Shannong 19	63.57	71.27	66.14	64.49
L190	Shannong 06-278	62.25	91.55	48.51	64.67
L191	Yangmai 18	66.06	60.93	55.30	62.01
L192	Ningmai 17	58.77	82.45	63.74	65.02
L193	Ningmai 13	72.13	55.13	47.93	61.06
L194	Zhenmai 168	61.02	55.96	57.28	60.94
L195	Yangmai 12	44.87	55.46	56.46	58.62
L196	Yangmai 14	60.11	71.52	53.64	62.40
L197	Huamai 5	87.93	45.61	50.83	62.28
L198	Yangmai 20	68.87	60.59	61.26	63.13
L199	Yangmai 21	63.07	50.00	50.00	59.45
L200	Zhengmai 379	78.16	69.53	56.79	64.96
L201	Zhengmai 05706	50.16	50.25	47.85	57.48
L202	Zhengmai 113	50.02	61.67	57.28	60.24
L203	Zhengmai 369	40.12	83.52	66.55	63.06
L204	Zhengmai 05871	62.25	68.21	62.58	63.44

L205	Xinmai 19	43.23	55.13	62.00	59.09
L206	Shannong 26	52.32	59.27	56.95	60.18
L207	Shijiazhuang 8	79.88	63.41	65.40	65.52

YY, SQ, and KF are Yuanyang 2016, Shangqiu 2016 and Kaifeng 2016 in Yellow and Huai wheat region, respectively.

The best linear unbiased predictions (BLUP) values calculated from all three environments averaged phenotypic values.

Table S2. Marker-trait associations (MTAs) for Zn accumulation in the associated population analyzed by the mixed linear model (MLM).

**Environment** [1]	**SNP marker** [2]	**Chromosome**	**Physical position (bp)**[3]	***P*-value**	R^2 **(%)**[4]
KF	AX-111023583	1A	574477246	8.29E-05	9.29
KF	AX-110606195	1A	574477470	3.27E-05	10.26
KF	AX-110973606	1A	574479186	8.11E-05	9.32
KF	AX-110399529	1B	665798565	4.65E-05	9.89
KF	AX-110597902	1B	665800702	8.11E-05	9.32
KF	AX-111132092	1B	665823297	4.65E-05	9.89
KF	AX-110144700	1B	666010880	8.11E-05	9.32
KF	AX-110162520	1B	667883904	1.42E-05	11.14
KF	AX-110403620	1B	667911274	2.21E-06	13.12
KF	AX-109537654	1B	667963795	9.13E-05	9.19
KF	AX-109860530	1B	667979199	8.51E-06	11.68
KF	AX-111144645	1B	668002032	2.69E-07	15.44
KF	AX-109370201	1B	668002592	2.49E-05	10.54
KF	AX-89697158	1B	668050653	2.49E-05	10.54
KF	AX-110568620	1B	668073653	2.21E-06	13.12

KF	AX-109309348	1B	668078554	1.42E-05	11.14
KF	AX-109279194	1B	668083294	1.42E-05	11.14
KF	AX-109280602	1B	668084255	2.21E-06	13.12
KF	AX-110921776	1B	668098109	3.91E-05	10.07
KF	AX-110398589	1B	668098441	3.91E-05	10.07
KF	AX-111628012	1B	668100871	3.91E-05	10.07
KF	AX-110828223	1D	478184386	8.47E-05	9.27
KF	AX-109490599	2B	787099530	5.82E-05	9.66
KF	AX-95178106	2D	519133490	9.88E-05	9.11
KF	AX-94466886	2D	650654168	2.75E-05	10.44
KF	AX-111589110	3A	219103987	4.80E-05	9.86
KF	AX-108885346	3A	230193270	4.80E-05	9.86
KF	AX-108914831	3A	235844032	4.80E-05	9.86
KF	AX-110931111	3A	246635552	4.80E-05	9.86
KF	AX-110953100	3A	260492627	4.80E-05	9.86
KF	AX-94729264	3D	40526440	1.24E-06	13.76
KF	AX-108912427	4A	699571654	4.47E-07	14.87
KF	AX-110921340	5A	549409584	1.57E-05	11.02

KF	AX-109425582	5A	549409916	1.32E-05	11.21
KF	AX-108877412	5A	549426151	1.15E-05	11.35
KF	AX-109827457	5A	549427496	1.15E-05	11.35
KF	AX-111579944	5A	549427917	7.05E-06	11.88
KF	AX-111463331	5A	549576304	4.61E-06	12.33
KF	AX-89328151	5A	549576546	5.57E-06	12.13
KF	AX-111212215	5A	549900744	4.59E-05	9.91
KF	AX-110374026	5A	550152893	8.76E-05	9.24
KF	AX-110471080	5A	550179282	2.43E-05	10.57
KF	AX-108894206	5A	550249192	7.70E-05	9.37
KF	AX-110657845	5A	550251710	7.93E-05	9.34
KF	AX-109915056	5A	551500060	3.64E-05	10.15
KF	AX-94405132	5A	552055922	3.82E-05	10.10
KF	AX-108806757	5A	552068639	2.43E-05	10.57
KF	AX-110464094	5A	552073099	2.43E-05	10.57
KF	AX-110395949	5A	552076065	2.43E-05	10.57
KF	AX-110370070	5A	552076128	2.43E-05	10.57
KF	AX-111674434	5A	552103464	6.63E-06	11.94

KF	AX-111086828	5A	552155591	1.12E-05	11.38
KF	AX-108818847	5A	552159946	2.43E-05	10.57
KF	AX-86178455	5A	552162031	4.56E-05	9.91
KF	AX-110067131	5A	552208450	2.82E-05	10.41
KF	AX-112289745	5B	78708064	2.47E-06	13.00
KF	AX-110443373	5B	693033900	8.15E-06	11.72
KF	AX-94961930	6A	613482310	1.72E-05	10.93
KF	AX-109538092	6B	708670301	2.09E-05	10.73
KF	AX-94702817	6B	708943077	3.66E-05	10.14
KF	AX-95210102	6B	708943119	6.47E-05	9.55
KF	AX-111660994	6B	712117440	8.83E-05	9.23
KF	AX-111149663	6B	712475640	2.35E-05	10.60
KF	AX-110536000	6D	464120129	5.33E-05	9.75
SQ	AX-110529533	1D	16132987	1.46E-05	12.64
SQ	AX-94598102	2D	1638170	9.19E-05	10.70
SQ	AX-110922471	3B	376625452	2.09E-06	14.75
SQ	AX-94729264	3D	40526440	4.45E-10	24.55
SQ	AX-108851891	4A	669454046	5.27E-05	11.28

SQ	AX-108912427	4A	699571654	1.14E-09	23.42
SQ	AX-89748062	4B	13996819	1.81E-05	12.42
SQ	AX-110931014	5A	650240330	9.82E-07	15.59
SQ	AX-112289745	5B	78708064	1.55E-05	12.58
SQ	AX-111012263	7A	261687749	8.04E-06	13.28
YY	AX-108995328	1A	592315138	3.55E-05	12.27
YY	AX-110038787	1B	564909314	1.03E-05	13.53
YY	AX-110620516	2B	154930484	8.62E-05	11.37
YY	AX-94583825	2D	582025967	5.40E-05	11.84
YY	AX-110922471	3B	376625452	5.69E-08	19.07
YY	AX-94567805	3B	779542533	7.58E-05	11.50
YY	AX-94729264	3D	40526440	3.69E-10	24.77
YY	AX-108912427	4A	699571654	1.92E-09	22.87
YY	AX-110931014	5A	650240330	1.08E-06	15.89
YY	AX-112289745	5B	78708064	3.29E-07	17.16
YY	AX-111084964	6B	462555585	5.22E-05	11.88
YY	AX-111012263	7A	261687749	3.21E-06	14.74
BLUP	AX-110038787	1B	564909314	4.33E-05	13.78

BLUP	AX-94729264	3D	40526440	2.32E-05	14.40
BLUP	AX-111858412	3D	515115709	3.18E-05	14.08
BLUP	AX-109319838	3D	516391195	9.13E-05	13.04
BLUP	AX-109799345	3D	519527578	8.69E-05	13.09
BLUP	AX-108912427	4A	699571654	8.02E-05	13.17
BLUP	AX-94932868	5A	42281607	6.31E-05	13.41
BLUP	AX-110931014	5A	650240330	3.39E-05	14.02
BLUP	AX-109482930	5B	57158679	4.45E-05	13.75
BLUP	AX-110555735	5B	57445997	4.45E-05	13.75
BLUP	AX-110398218	5B	57493343	3.92E-05	13.87
BLUP	AX-111510481	5B	407053365	7.69E-05	13.21
BLUP	AX-109965599	5B	407323613	7.69E-05	13.21
BLUP	AX-109842469	5B	409148404	6.13E-05	13.43
BLUP	AX-111529353	5B	409473826	8.12E-05	13.16
BLUP	AX-110441788	5B	410547248	8.82E-05	13.08
BLUP	AX-108899168	5B	410604227	8.36E-05	13.13
BLUP	AX-109371460	5B	410880297	6.85E-05	13.32
BLUP	AX-109817251	5B	410880846	4.81E-05	13.67

BLUP	AX-109654495	5B	410880947	4.81E-05	13.67
BLUP	AX-111756090	5B	410881074	4.81E-05	13.67
BLUP	AX-110925919	5B	410949343	4.81E-05	13.67
BLUP	AX-110054307	5B	410977495	4.81E-05	13.67
BLUP	AX-109451599	5B	410983127	4.81E-05	13.67
BLUP	AX-110673781	5B	411005244	4.56E-05	13.72
BLUP	AX-110548546	5B	411019312	4.81E-05	13.67
BLUP	AX-109891068	5B	411022935	4.81E-05	13.67
BLUP	AX-108780883	5B	411079760	4.81E-05	13.67
BLUP	AX-110374478	5B	411080602	4.81E-05	13.67
BLUP	AX-108960031	5B	411085971	4.81E-05	13.67
BLUP	AX-111211356	5B	411091783	4.65E-05	13.71
BLUP	AX-108894319	5B	411220984	4.81E-05	13.67
BLUP	AX-111586134	5B	411309520	4.81E-05	13.67
BLUP	AX-110674497	5B	411322971	5.22E-05	13.59
BLUP	AX-109469061	5B	411324833	4.81E-05	13.67
BLUP	AX-109957391	5B	411355893	5.79E-05	13.49
BLUP	AX-108836943	5B	411505891	4.81E-05	13.67

BLUP	AX-108915702	5B	411544100	4.60E-05	13.72
BLUP	AX-111062161	5B	411544224	4.81E-05	13.67
BLUP	AX-111737326	5B	411544316	5.76E-05	13.50
BLUP	AX-111102604	5B	411621476	4.81E-05	13.67
BLUP	AX-111492166	5B	411623575	4.81E-05	13.67
BLUP	AX-108940698	5B	411623696	4.81E-05	13.67
BLUP	AX-108985291	5B	411623737	4.81E-05	13.67
BLUP	AX-111622694	5B	411638915	5.46E-05	13.55
BLUP	AX-109308004	5B	411649815	4.81E-05	13.67
BLUP	AX-110384837	5B	411654474	4.81E-05	13.67
BLUP	AX-109335293	5B	411753477	4.81E-05	13.67
BLUP	AX-109471499	5B	411785464	4.81E-05	13.67
BLUP	AX-89548536	5B	411799172	4.81E-05	13.67
BLUP	AX-86176241	5B	411929890	3.15E-05	14.09
BLUP	AX-111775333	5B	412175187	5.61E-05	13.52

[1] Phenotypic values collected from three locations (KF, 2016 Kaifeng; SQ, 2016 Shangqiu; YY, 2016 Yuanyang), and the best linear unbiased predictions (BLUP) values calculated from all three environments.

[2] Markers were detected at the threshold -log10 (P) equaling 4.0.

[3] P-value of the corresponding significant SNPs calculated by MLM model.

[4] R^2 of Model with significant SNP.
